# Combined Vaccination with Recombinant HVT-ND and Live Newcastle Disease Vaccines Accelerates Early Immunity, Limits Virus Shedding and Reduces Transmission

**DOI:** 10.3390/vaccines14100859

**Published:** 2026-09-28

**Authors:** Edit Kovács, Tímea Tatár-Kis, Balázs Felföldi, Zalán G. Homonnay, István Kiss, Krisztián Bányai, Vilmos Palya

**Affiliations:** 1 Ceva-Phylaxia Ltd., 1107 Budapest, Hungary; edit.kovacs@ceva.com (E.K.); timea.tatar-kis@ceva.com (T.T.-K.); balazs.felfoldi@ceva.com (B.F.); zalan.homonnay@ceva.com (Z.G.H.); istvan.kiss@ceva.com (I.K.); 2 Department of Medical Biology, Medical School, University of Pécs, 7624 Pécs, Hungary; bkrota@hotmail.com; 3 Department of Pharmacology and Toxicology, University of Veterinary Medicine, 1078 Budapest, Hungary

**Keywords:** Newcastle disease, genotype VII.2 NDV, vaccination program, efficacy, virus shedding, transmission

## Abstract

**Background/Objectives:** Effective control of Newcastle disease (ND) requires a reduction in virus shedding and transmission. While recombinant herpesvirus of turkey vectored Newcastle disease (rHVT-ND) vaccines induce strong systemic immunity, their ability to generate early mucosal immunity and limit transmission may not be optimal when used alone. **Methods:** Day-old layer chicks with maternally derived antibodies were vaccinated with the rHVT-ND vaccine alone or in combination with conventional live ND vaccines administered either at hatch or in a booster program. Birds were challenged with a genotype VII.2 velogenic ND virus strain at 3, 4 or 6 weeks of age. Humoral immune responses, clinical protection, challenge virus shedding and transmission to vaccinated contact groupmates were assessed. **Results:** All vaccination programs provided strong clinical protection (85–100%); however, combined vaccination strategies induced earlier immunity and reduced virus shedding more efficiently than the rHVT-ND vaccine alone. Booster vaccination further accelerated the onset of immunity and decreased shedding. By 6 weeks of age, differences among vaccination regimens had mainly disappeared, with all vaccinated groups showing minimal or no challenge virus shedding. Transmission to vaccinated contact birds was significantly reduced in the combined vaccinated groups, whereas unvaccinated controls efficiently spread the challenge virus and consequently caused high mortality in the contact groupmates. **Conclusions:** Both the rHVT-ND vaccine alone and in combination with live ND vaccines provided similar clinical protection against genotype VII.2 NDV challenge; however, combined vaccination programs accelerated immunity and more effectively reduced virus shedding and transmission, supporting their use for improved ND control, particularly in endemic regions.

## 1. Introduction

Newcastle disease (ND) remains one of the most significant avian viral diseases globally, posing a persistent threat to the poultry industry [1,2]. It is caused by *Orthoavulavirus javaense* (formerly *Avian orthoavulavirus 1* or APMV-1), commonly referred to as Newcastle disease virus (NDV), within the *Paramyxoviridae* family. Even though it belongs to one single serotype, NDV has undergone significant genetic diversification, with virulent genotype VII strains currently responsible for many outbreaks worldwide and posing serious challenges to existing control strategies [3].

NDV infection can manifest in various forms ranging from subclinical infections to devastating outbreaks, with mortality rates of up to 100% in susceptible flocks [2,4]. While vaccination has been the cornerstone of ND control since the 1950s, the virus continues to circulate and evolve, often causing “silent” infections in vaccinated birds that lack sterilizing immunity [5]. Effective control of ND requires not only the prevention of clinical disease but also reductions in virus shedding and transmission within and among flocks.

Conventional live ND vaccines, including lentogenic strains, such as the LaSota, HB1, PHY.LMV.42, V4 or I2 strains, have been widely used for decades to control ND outbreaks. The critical limitation of traditional NDV vaccines is their inability to prevent infection and subsequent viral shedding, especially following challenge with recent velogenic field NDV strains [6,7,8]. The basis for reduced protection against the recently circulating virulent field NDV strains is unclear. Some scientists suggest that antigenic mismatch between the vaccine and the circulating field virus may help the field virus to evade traditional vaccine-induced immunity. Indeed, different research groups have shown that the ND vaccine, which is homologous to the challenge virus genotype, is more potent in reducing challenge virus shedding than a vaccine of a heterologous genotype [6,9]. Other scientists suspect that the quality (precision) of vaccine application, or concomitant infection with other agents that compromise the immune responsiveness of the chickens in the field, are responsible for vaccination failures [7,10,11]. It has also been established that the hemagglutination inhibition (HI) antibody titer that is induced by the conventional vaccines correlates well with protection, irrespective of the genotypes of the vaccine and challenge strains [7,12]. It should also be noted that interference by maternally derived antibodies (MDAs) can compromise the efficacy of both live and killed vaccines administered at hatch or early in life. These limitations are particularly pronounced in endemic areas, where constant exposure to infection from young age necessitates early onset of robust immunity.

Recent advancements in vaccine technology and administration methods have focused on bypassing maternal antibodies’ interference with vaccination at the hatchery (either by in ovo application, or by subcutaneous injection at a day old) and enhancing mucosal immunity to prevent infection and limit virus shedding and transmission. Recombinant turkey herpesvirus-vectored ND vaccines (rHVT-ND) expressing the NDV fusion (F) protein have been developed to address some of these challenges. rHVT-ND vaccines provide systemic and long-lasting immunity and are only mildly affected by maternally derived antibodies. Previous studies, however, have shown that when rHVT-ND vaccines are administered alone, the clinical protection and the suppression of virus shedding, mainly by the respiratory route, may not be optimal in the first two-three weeks of life [13,14,15], despite these being highly necessary in areas where ND is endemic [13,16,17].

To overcome these limitations, combined vaccination strategies using rHVT-ND in conjunction with conventional live ND vaccines at a day old, or in a prime–boost regimen, have been investigated. Combining rHVT-ND vaccination with live ND vaccines may offer complementary advantages, including earlier onset of immunity and enhanced mucosal protection. It is very much required in areas where ND is endemic to take advantage of the rapid induction of mucosal immunity elicited by live ND vaccines alongside with the long-term systemic and cell-mediated immunity facilitated by rHVT-ND [14,17,18,19]. This may be particularly important under conditions of high infection pressure, where early protection is critical. Today, however, limited data are available regarding the impact of such combined vaccination strategies on the transmission of field NDV within vaccinated flocks.

Studies have shown that simultaneous administration of rHVT-ND and live ND vaccines increases protection and antibody levels compared with single-vaccine protocols, as measured by hemagglutination inhibition (HI) and the enzyme-linked immunosorbent assay [19,20,21,22]. In addition, challenge studies demonstrate that prime–boost regimens significantly reduce virus shedding [14], which is critical for interrupting transmission following infection with virulent genotype VII NDV strains.

Despite these promising observations, well-controlled challenge studies directly comparing multiple rHVT-ND-based vaccination regimens, including combined vaccination with conventional live vaccines in prime or prime-boost programs, remain limited. Systematic evaluation of humoral immune responses, virus shedding and transmission dynamics following challenge with recent dominant field velogenic NDV is needed to optimize vaccination strategies in endangered poultry flocks.

The present study aimed at evaluating the efficacy of an rHVT-ND vaccine applied alone or as part of combined vaccination programs with live ND vaccines in commercial layer chickens carrying MDA to NDV. Specifically, the study investigated the effects of these vaccination strategies on clinical protection, virus shedding, and consequent transmission following challenge with a velogenic genotype VII.2 NDV strain at different time points after vaccination.

## 2. Materials and Methods

### 2.1. Vaccines and Challenge Virus

Cryo-preserved cell-associated rHVT-ND vaccine (Vectormune^®^ ND, Ceva-Phylaxia, Budapest, Hungary) expressing the F protein of the avirulent D26/76 genotype I NDV strain (GenBank accession number: M24692) was used in this study. The vaccine was diluted in the corresponding vaccine diluent to obtain one commercial dose in 200 µL, which was administered by the subcutaneous route.

Three types of conventional live NDV vaccine were used: (1) a live enterotropic NDV vaccine containing the PHY.LMV.42 strain from genotype I (Cevac^®^ Vitapest L vaccine, Ceva-Phylaxia Ltd., Budapest, Hungary-referred to as enterotropic ND vaccine); (2) live respiratropic vaccine composed of the Hitchner B1 strain (abbreviated as HB1 vaccine) from genotype II (Cevac^®^ Uni L); and (3) a live respiratropic vaccine containing the LaSota strain (referred to as LaSota vaccine) from genotype II (Cevac^®^ New L). The conventional live ND vaccines were administered by the intranasal route using one commercial dose in 20 µL.

The challenge virus strain was a genotype VII.2 viscerotropic velogenic NDV (vNDV, isolate id.: D3273/1/16/TR; GenBank accession number of the fusion protein gene: MT876630), a sub-genotype which is highly prevalent in current outbreaks worldwide. This strain induces 100% mortality between 4 and 5 days post-challenge in 3-week-old specified pathogen-free (SPF) chickens following intranasal inoculation at a dose of 5.0 log_10_ ELD_50_. The challenge virus was diluted in sterile phosphate-buffered saline (PBS) to obtain 5.0 log_10_ ELD_50_ in a dose of 100 µL.

### 2.2. Animals and Experimental Design

The experimental design is presented in Table 1. A total of five hundred and ninety-five, day-old commercial brown layer chicks, possessing maternally derived antibodies (MDA) against NDV were purchased from a commercial hatchery in Hungary (breed: Tetra SL, Uraiújfalu, Bábolna Tetra Ltd., Bábolna, Hungary). Blood samples were taken from 20 chicks on the day of hatch to determine the MDA levels against NDV. Ten specified pathogen-free (SPF) chickens were included as controls for the first challenge date to validate the pathogenicity of the challenge virus (Group 6). Before the challenges, the birds were housed in isolated experimental units (BSL-2) under standard husbandry conditions; for the challenges, they were moved to a BSL3 unit.

On arrival, the day-old chicks were allocated into experimental groups, designated as Group 1 to 6, receiving different vaccination regimens, all of which, except group 1 and 6, included the administration of rHVT-ND vaccine. In the selected groups, live ND vaccines were administered via the nasal route at day old, and in one group, an additional booster vaccination was applied at 12 days of age. Throughout the study, blood samples were collected from 20 randomly selected non-challenged chickens per group at each sampling time point to monitor the decay of maternally derived antibodies (MDAs) and the antibody response to vaccination until 8 weeks of age.

Birds receiving the same vaccination regime were allocated into three subgroups of chickens being challenged at 3, 4 or 6 weeks of age. During the post-vaccination/pre-challenge period (till 3, 4 or 6 weeks of age), each group of chickens was housed in separate, isolated BSL-2 animal rooms.

To evaluate both the challenge virus shedding and the transmission dynamics, a controlled-contact infection model was implemented at each challenge time point. Transmission of the challenge virus was assessed by co-mingling contact groupmates with the directly challenged birds and monitoring virus shedding and serological responses both in seeders and contacts. Before the challenges, at 3, 4 or 6 weeks of age, each subgroup was assigned into two further subgroups: (i) a seeder subgroup (*n* = 20 chickens) and (ii) a contact subgroup (*n* = 10 chickens). Seeder subgroups were transferred to a BSL-3 facility first and challenged intranasally with a dose of 5.0 log_10_ELD_50_ of the vNDV challenge strain. Twenty-four hours post-challenge, 10 non-challenged birds from the same experimental group (contact birds) were introduced into the same enclosure to assess the challenge virus’ transmission from seeders to contact birds. The ratio of directly challenged seeders to contact chickens was 2:1. This design was used to simulate a high-infection-pressure scenario within a flock. Transmission was evaluated over a 13-day post-contact period, using both the virus shedding and serological monitoring results. Oronasal (ON) and cloacal (CLO) swabs were collected from the seeder chickens on the 5th day post-challenge (dpch), and from the comingled, contact chickens on the 5th aand 14th dpch. Sterile EUROTUBO^®^ Collection swabs were used (code: 300203; Deltalab, Rubi Barcelona, Spain); dry swab samples were stored at −80 °C until processing. Blood was collected before the challenge from all birds included in the challenge infection and at the end of the post-challenge observation period from each chicken that survived. A challenged bird was considered protected if it showed no ND-specific clinical signs (i.e., depression, ruffled feathers, respiratory distress, nasal-ocular discharges, diarrhea, nervous signs like paralysis, torticollis) or mortality during the 14-day post-challenge observation period.

The humane endpoint was determined as the point at which a severely diseased chicken could not eat or drink any longer (euthanized chickens were counted among the mortalities on the day of euthanasia). Chickens reaching the humane endpoint and all surviving birds at the end of the post-challenge observation period were euthanized by sodium pentobarbital (450 mg/bodyweight kg).

### 2.3. Detection of Vaccine Take

Feather pulp from axillary feathers and spleen was collected from ten birds per vaccinated group and from five non-vaccinated layers at 21 days post vaccination (dpv). Replication of the rHVT-ND vaccine virus was assessed using a real-time PCR assay targeting the *SORF1* gene [19], capable of detecting both HVT and recombinant HVT viruses. The presence of HVT DNA either in the spleen or feather pulp or both collected from the same bird was considered indicative of successful vaccine take.

### 2.4. Monitoring of Antibody Response to Vaccination and Challenge

The decay of MDA and the humoral immune response to vaccination were monitored through regular blood sampling from randomly selected birds up to 8 weeks of age (D0, w2, w3, w4, w5, w6 and w8).

To check the possible booster effect of challenge infection on the antibody response to NDV in the vaccinated birds, blood samples were collected before each challenge, and at the end of the 14-day-long post-challenge (pch) observation period. Antibody levels against NDV were measured both by the hemagglutination inhibition (HI) test and with a commercial ELISA (ID Screen^®^ Newcastle Disease Indirect ELISA Kit, Product code: NDVS, IDVet, Grabels, France), designed for sensitive detection of antibody responses to rHVT-ND vaccines.

The HI test was carried out using 4 HAUs of NDV LaSota antigen according to the World Organization for Animal Health (WOAH, formerly OIE) Terrestrial Manual [23], with the modification that 50 µL of PBS, serum, antigen, or red blood cell suspension was used instead of 25 µL. A threshold of more than a 2 log_2_ (4-fold) increase in the HI titer in the contact birds during the post-challenge observation period was considered indicative of infection by the challenge virus [24]. The ELISA test was performed according to the manufacturer’s instructions.

### 2.5. Quantification of Challenge Virus Shedding

Individual swab samples were eluted in 1 mL sterile PBS with Tissue Lyser^®^. Then, samples were clarified by centrifugation (900× *g* for 3 min at room temperature). RNA was extracted from the supernatant with the Cador Pathogen 96 QIACube HT kit using the QIACube HT device (QIAGEN) according to manufacturer’s instructions. A one-step quantitative RT-real-time PCR (RT-qPCR) assay targeting the matrix (M) gene of NDV [25] was performed. Cycle threshold (Ct) values were used to compare the challenge virus load in the swabs. To align the different runs, three steps of the tenfold dilution series of TaqMan™ NDV RNA Control (Thermo Fisher Scientific, Waltham, MA, USA) were prepared, aliquoted and included in all runs. Samples with Ct values below 40 were considered positive.

### 2.6. Assessment of Transmission

Transmission was evaluated based on detection of NDV RNA and the HI antibody titer increase in the contact birds. For group-level evaluation of the HI titer increase, post-challenge results were compared to their non-challenged groupmates at the same age. Birds were considered protected against detectable infection (i.e., no challenge virus transmission) if they did not shed a detectable amount of ND virus and did not show a significant increase in HI antibody titers (defined as ≤2 log_2_ increase) during the 2-week post-challenge observation period.

### 2.7. Statistical Analysis

For the proportions of clinically protected birds, RT-qPCR positivity in oral and cloacal swab samples collected after challenge, and birds with a serological immune response to the challenge, 95% confidence intervals were calculated using the Wilson/Brown method; NDV load in oronasal and cloacal swabs was compared based on Ct values using GraphPad Prism software version 10.0.2 (GraphPad Software Inc., Boston, MA, USA). The same types of samples (i.e., either oronasal or cloacal swabs) were compared among the groups challenged at the same age using the Kruskal–Wallis test followed by Dunn’s multiple comparisons. Statistical significance was set at *p* < 0.05.

## 3. Results

### 3.1. Vaccine Take and Replication of rHVT-ND

Replication of the rHVT-ND vaccine virus was checked and confirmed at 21 days of age. Vaccine virus DNA was detected in all vaccinated birds, based on analysis of feather pulp and spleen samples (Table 2). These results confirm consistent vaccine uptake and vaccine-virus replication across all vaccinated groups. The non-vaccinated control chickens remained negative.

### 3.2. Decay of Maternally Derived Antibodies Against NDV and Humoral Immune Response to Vaccination

**Figure 1:**
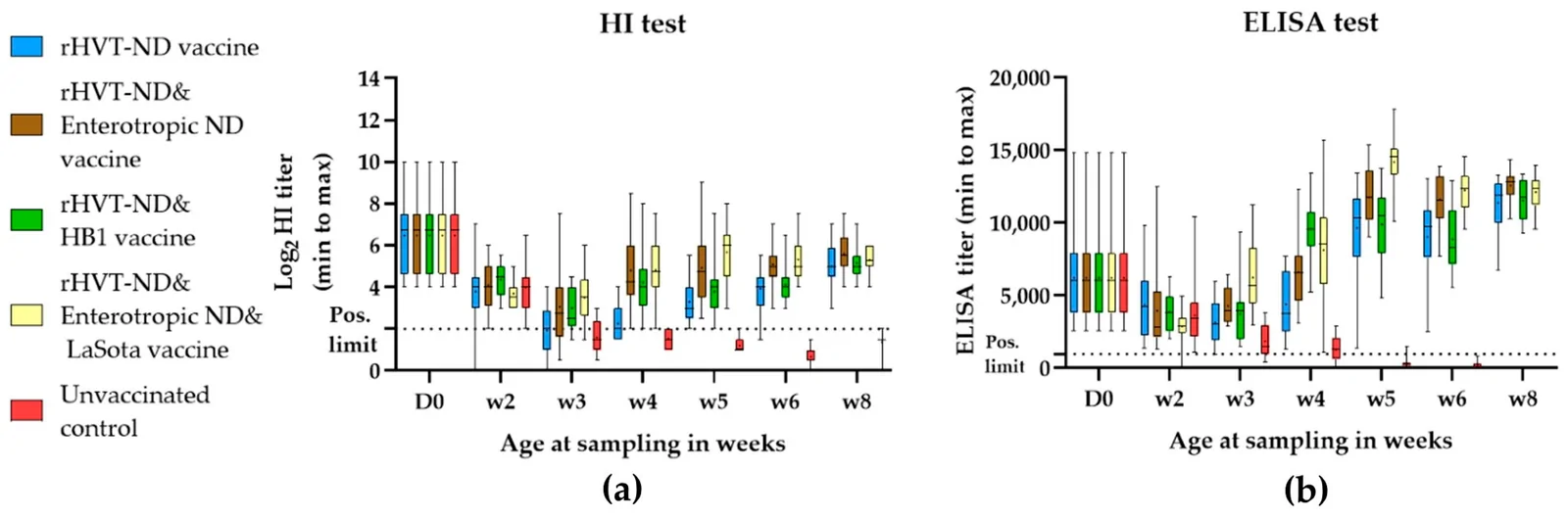
Changes in NDV-specific antibody titers measured in the serum samples of the commercial layer chickens with (**a**) HI and (**b**) ELISA (IDScreen^®^ NDV Indirect ELISA) methods. The cross within each box plot represents the group mean, while the horizontal line indicates the median.

At hatch, the chicks had a mean HI titer of 6.4 ± 1.2 log_2_ and an ELISA titer of 5973 ± 3209 (mean ± SD). The decay of MDA showed a steady decline and dropped below the positivity limit between 21 and 28 days of age in the unvaccinated control group. In the vaccinated groups, a progressive decline in HI titers was observed during the first three weeks of life, which was followed by a sustained increase in titers during the coming weeks. The increase in antibody titers was first detectable compared to the unvaccinated controls (G1) at 3 weeks of age in groups receiving combined vaccination, whereas in the rHVT-ND-only group (G2), this occurred at 4 weeks of age. The increase in HI titer, compared to that on the previous sampling date, was seen first at 4 weeks of age; regardless the vaccination regimen was the slower decrease in MDA titers compared to that in the unvaccinated group. The group that received a booster vaccination (G5) demonstrated a faster antibody rise, compared to non-boosted groups. Despite these initial differences in the onset of antibody response, all vaccination programs reached similar levels of antibody titers by 8 weeks of age (Figure 1a, Table S4). In detecting vaccine-induced antibody titer increases earlier, ELISA demonstrated higher sensitivity, but a similar kinetic profile, compared to the HI test. This finding was consistent in all vaccinated groups, including the rHVT-ND-only group as well (Figure 1b).

### 3.3. Clinical Protection Against Velogenic NDV Challenge

**Figure 2:**
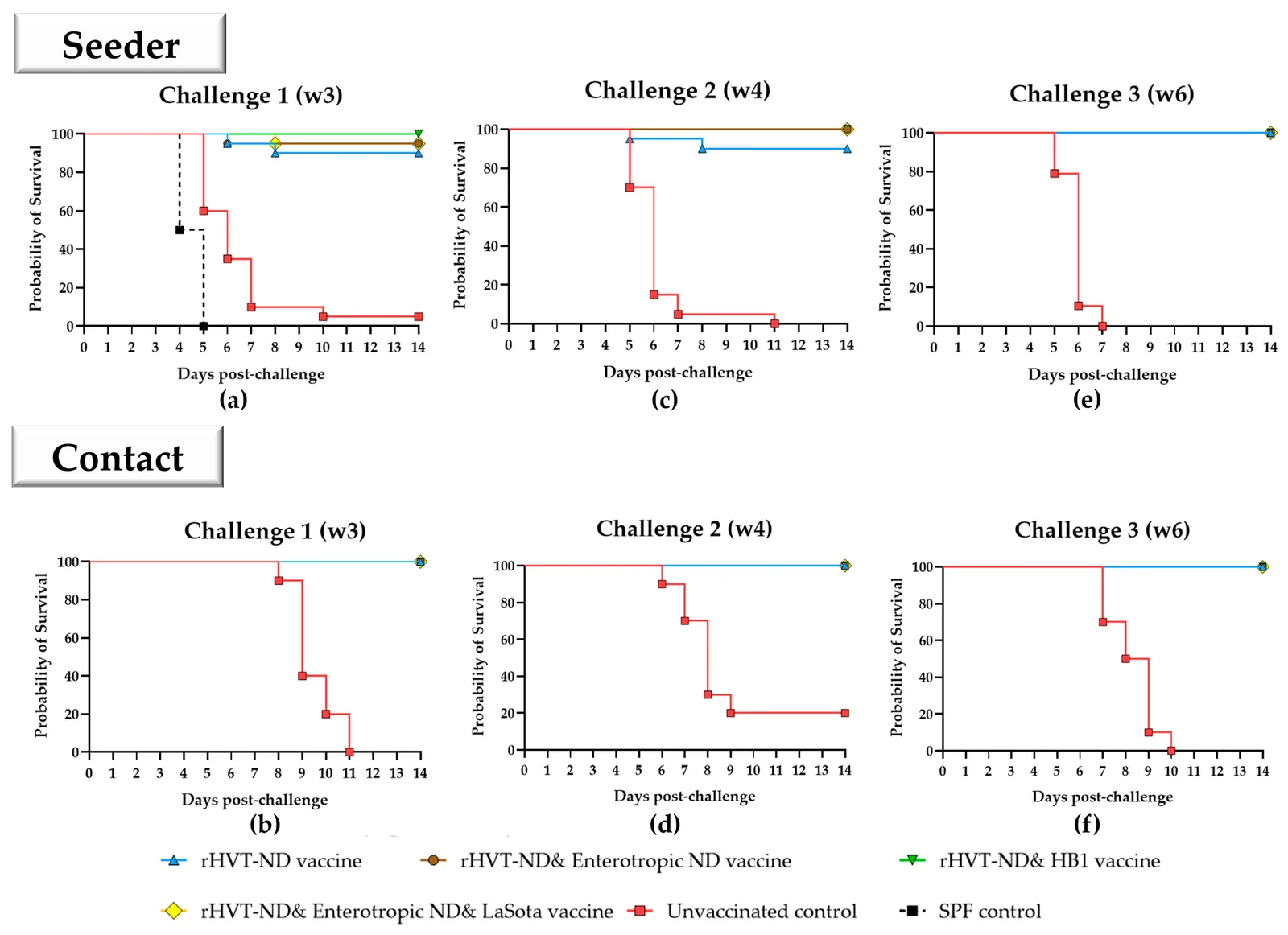
Survival of seeder birds (**a**) challenged at 3, (**c**) 4 and (**e**) 6 weeks of age and survival of contact animals challenged at (**b**) 3, (**d**) 4 and (**f**) 6 weeks of age.

All non-vaccinated SPF chickens succumbed to the challenge between 3 and 5 dpch. This result validates the high virulence of the challenge virus and the infection method. Non-vaccinated commercial layers inoculated with the challenge virus demonstrated 95% mortality at 3 weeks of age; the survival of one bird was most probably due to the presence of some residual MDA at this age. In later-age challenges, all non-vaccinated control birds developed severe clinical signs and died within 4–7 days post-challenge. In contrast, all vaccination regimens provided a high level of clinical protection against the challenge at all tested ages (Table 3, Figure 2a,c,e). At three weeks of age, clinical protection in directly challenged birds ranged from 90% to 95% in groups receiving combined vaccination, while slightly lower protection (85%) was observed in the rHVT-ND-only group. At 4 weeks of age, protection improved further, reaching 100%, except in the rHVT-ND-only group, where protection remained at 85%. By 6 weeks of age, 100% clinical protection was recorded in all vaccinated groups.

Vaccinated contact birds remained clinically healthy regardless of the vaccination regimen or age at exposure to the challenge (Table 3, Figure 2b,d,f). Conversely, contact birds in the non-vaccinated control groups developed disease and exhibited high mortality (80–100%), confirming efficient transmission of the challenge virus to contact birds under these experimental conditions.

### 3.4. Virus Shedding of Directly Challenged and Contact Birds

**Figure 3:**
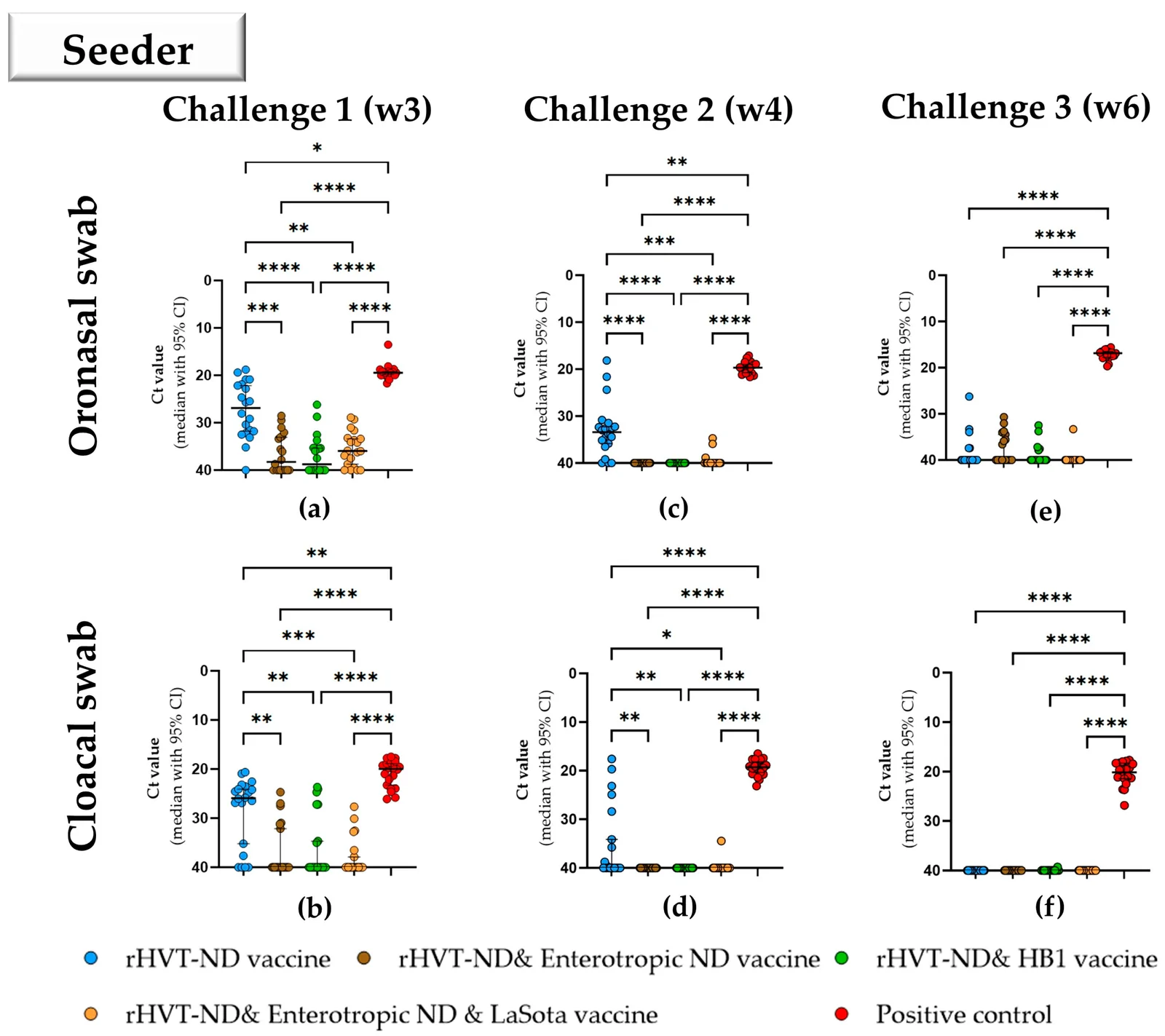
Virus shedding of direct-challenged birds (seeders) at 5 dpch at different ages. (**a**) Oronasal and (**b**) cloacal shedding after challenge at 3 weeks of age; (**c**) oronasal and (**d**) cloacal shedding after challenge at 4 weeks of age; (**e**) oronasal and (**f**) cloacal shedding after challenge at 6 weeks of age. The asterisks indicate different levels of statistically significant difference; * means, *p* < 0.05, **: *p* < 0.01, ***: *p* < 0.001, ****: *p* < 0.0001.

**Figure 4:**
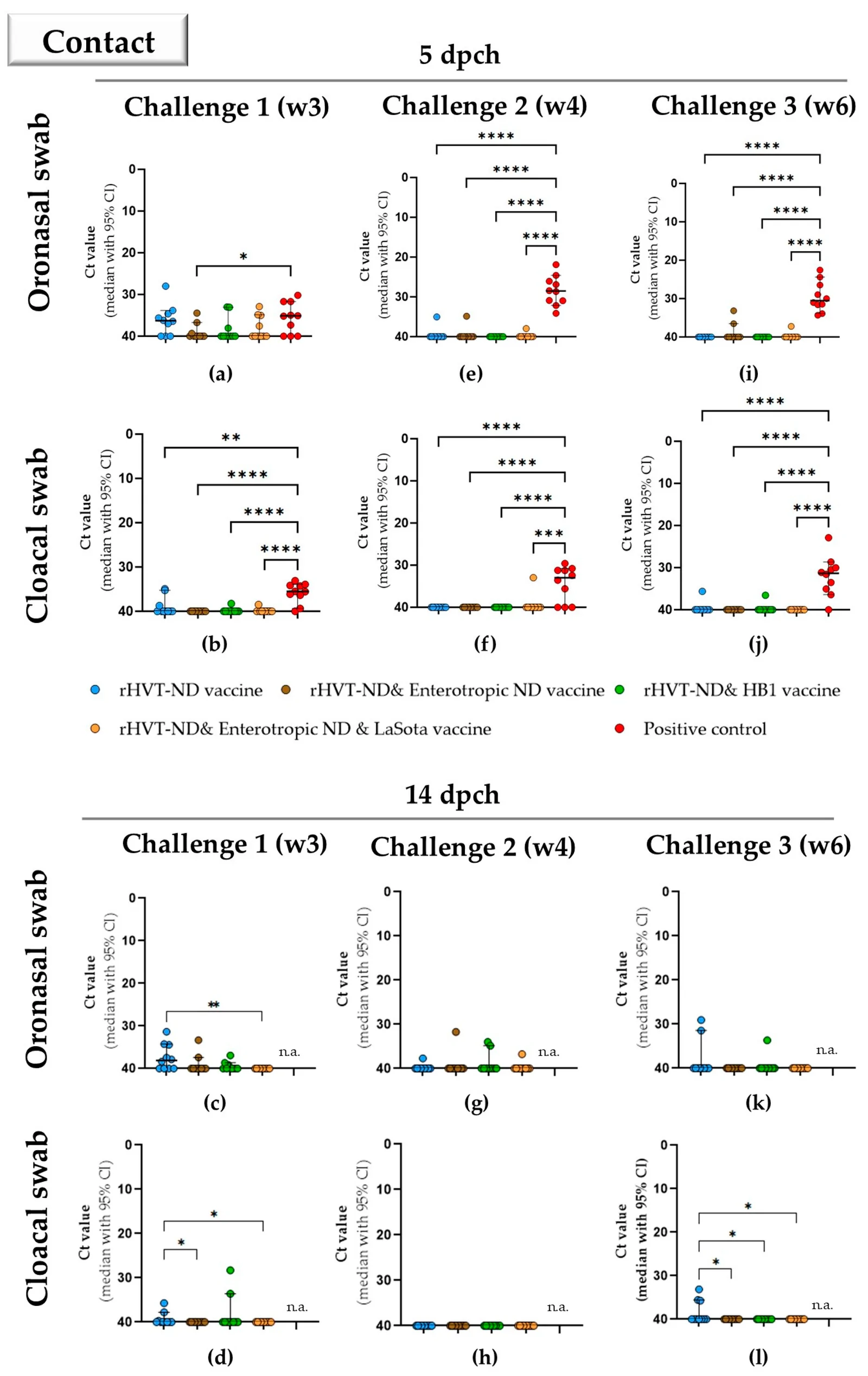
Virus shedding of contact subgroups based on oronasal and cloacal swab samples at 5 and 14 dpch (**a**–**d**) after challenge at 3 weeks of age, (**e**–**h**), 4 weeks of age (**i**–**l**) and 6 weeks of age. The asterisks indicate the statistically significant differences; * means *p* < 0.05, **: *p* < 0.01, ***: *p* < 0.001, ****: *p* < 0.0001. n.a.: not applicable (all the controls died before this sampling).

At 3 weeks, all vaccination regimens significantly reduced virus shedding in directly challenged birds compared to non-vaccinated controls. Groups receiving combined vaccination showed a markedly greater reduction in virus shedding than the rHVT-ND-only group. This effect was most evident in cloacal shedding, where 80% of birds in the rHVT-ND group excreted a measurable amount of the challenge virus, compared to only 30–35% in the other groups (Table 4, Figure 3a,b). In the vaccinated contact chickens, minimal or no NDV RNA could be detected across all groups at both sampling dates and sites (Figure 4a–d, Table S1).

After the challenge at 4 weeks of age, virus shedding was further reduced in the directly challenged groups. In the rHVT-ND group, although most samples yielded positive results by RT-qPCR, the viral loads were lower than those observed after the challenge at 3 weeks of age, suggesting improved suppression of challenge virus dissemination and replication. In all combined vaccination groups, no or a minimal amount of ND viral RNA was found both in the ON and CLO swab samples (Figure 3c,d, Table S2). In the contact birds, no or a negligible amount of NDV RNA could be detected across all combined vaccination groups (Figure 4e–h, Table S2).

At 6 weeks of age, the proportion of positive oronasal samples was low in all vaccinated groups, while all cloacal swabs were negative, indicating that the challenge virus could not disseminate systemically due to the development of a robust immunity that could prevent both infection and challenge virus shedding. (Figure 3e,f, Table S3). No or only a very low amount of NDV RNA was detected in contact birds across all combined vaccination groups (Figure 4i–l, Table S3).

Overall, statistical analysis confirmed that combined vaccination improved the suppression of virus shedding significantly compared to rHVT-ND vaccination alone at 3 and 4 weeks of age (*p* < 0.05). Differences among the vaccination regimens were no longer detectable at 6 weeks of age.

### 3.5. Humoral Immune Response to Challenge

**Figure 5:**
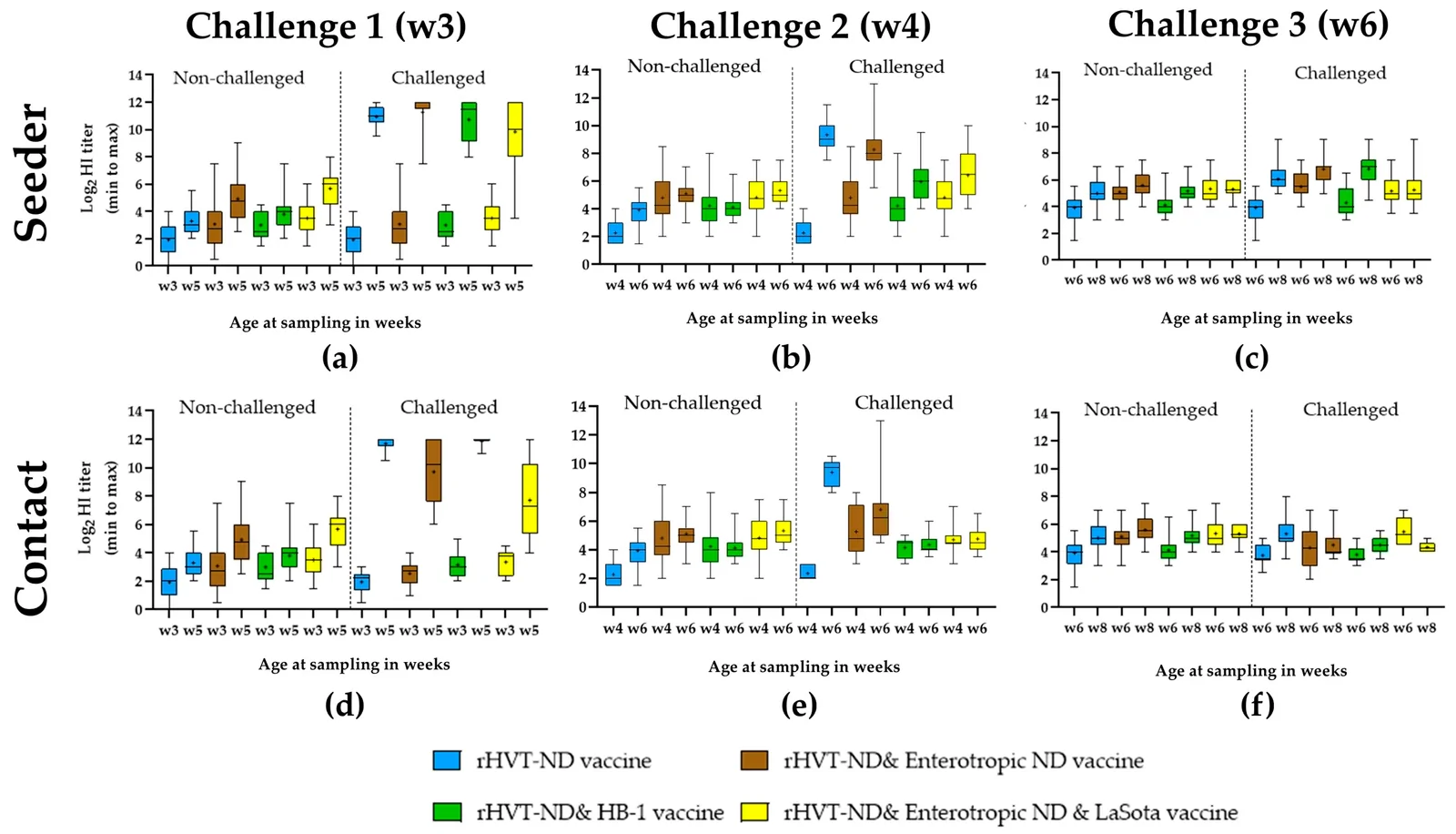
Antibody titres before and after challenge in vaccinated, challenge-exposed groups and age-matched non-challenged groups. Results are shown for seeder subgroups challenged at (**a**) 3, (**b**) 4, and (**c**) 6 weeks of age, and for contact subgroups challenged at (**d**) 3, (**e**) 4, and (**f**) 6 weeks of age. No results are presented for the positive control group due to 100% or near-total mortality. The cross within each box plot represents the group mean, while the horizontal line indicates the median.

Direct challenge infection at 3 weeks of age induced a marked increase in HI antibody titers in all vaccinated groups, indicating active viral replication and boosting of the primer immune response (Figure 5a, Table 5). This increase was less pronounced in the booster-vaccinated group, suggesting improved control of challenge virus replication. Following challenge at 4 weeks of age, the level of titer increase was restricted across all groups, with only the rHVT-ND group revealing a notable booster effect on the antibody titer, while combined vaccination groups manifested minimal antibody titer increase (Figure 5b). By 6 weeks of age, the booster effect of the challenge on the antibody titer was negligible in all vaccinated groups, with the rHVT-ND group showing comparable results to the combined vaccination programs (Figure 5c).

In the vaccinated contact birds, after comingling with their groupmates, challenged at 3 weeks of age, a marked HI antibody titer increase could be measured, indicating efficient transmission of the challenge virus from the directly challenged chickens (Figure 5d). After the challenges at 4 and 6 weeks of age, no increase or only a limited increase in antibody titers could be measured in the vaccinated contacts (Figure 5e,f). An exception was observed in the rHVT-ND-only contact subgroup at 4 weeks, where a moderate increase in titers could still be detected (Figure 5e). These findings are consistent with improved control of virus shedding and transmission at later ages and with the use of combined vaccination regimes.

### 3.6. Evaluation of Infection Status of Contact Chickens

To assess infection status in the contact birds, virus shedding data was combined with the data on serological responses. A bird was classified as (i) protected against detectable infection if NDV RNA was not detectable and the HI titer increased by ≤2 log_2_, and (ii) exposed/infected if NDV RNA was detectable and/or the HI titer increased by >2 log_2_. The cut-off for the serological evaluation was set based on the weekly follow up of unchallenged vaccinated groupmates. Results indicated that a small increase in HI titers (less than 2 log_2_) could be detected during the 2-week periods covering the post-challenge observation time. Due to the ongoing development of the humoral immune response to vaccination (mainly during the period after the first two challenges), antibody level increase during the post-challenge observation period could be induced by vaccination; therefore, a >2 log_2_ HI titer increase in the contact birds was not taken as an indication of infection, rather as the result of the ongoing development of an antibody response to vaccination.

In the non-vaccinated control groups, efficient transmission was confirmed, as all contact birds developed clinical signs indicative of ND and died. Due to mortality, complete longitudinal sampling was not possible in these groups.

After the challenge at 3 weeks of age, contact groupmates showed that only the booster-vaccination regimen could provide detectable protection against horizontal infection (no virus shedding and no booster effect on antibody titer) in 20% of birds (Table 6). Other vaccination regimens could not prevent transmission to vaccinated groupmates. Although the NDV amount measured in swab samples indicated only weak shedding, there was a detectable infection in all the contacts (either by RT-qPCR or serology). In this early immunity phase, the incomplete immunity still allowed transmission and consequent infection, which was detectable primarily through serological responses rather than measuring virus shedding (Figure 4a,d, Table S4). It should, however, be noted that sampling for virus shedding was conducted only on two sampling dates during the two-week observation period. Combined vaccination, especially with booster vaccination, reduced transmission probability to the vaccinated contacts better than rHVT-ND alone.

At 4 weeks of age, improved immunity resulted in significantly reduced transmission. No detectable NDV RNA could be detected in 78–100% of vaccinated contact birds across all combined vaccination groups (Figure 4e–h). By serology, a reduced frequency of HI titer increases greater than 2 log_2_ was found compared to the results after challenge at 3 weeks (Figure 5b,e). Approximately 50–78% of birds in the combined vaccination and booster groups were found without detectable infection, while the rHVT-ND-only contact group was still susceptible to infection: although virus shedding was undetectable at 5 and 14 dpch, all chickens had an antibody titer increase, indicating efficient challenge virus transmission.

At 6 weeks, vaccination-induced immunity resulted in a lack of infection detection in most birds. No detectable NDV RNA was found in 70–100% of the vaccinated contact birds in the vaccinated groups (Figure 4i–l). By serology, minimal or no significant HI titer increases were observed (Figure 5f). Based on the results of the evaluation method used, 70–90% of the vaccinated contact birds proved to be protected against infection in the combined vaccination groups, whereas in the rHVT-ND-only group, less than 60% of contacts were found to be protected against infection.

When evaluated across all time points, in parallel with the development of the immune response to ND vaccination by age, virus shedding in the directly challenged birds was more and more significantly reduced, and as a consequence, transmission of the challenge virus to vaccinated contact groupmates showed a decreasing tendency (Table 6). Combined vaccination resulted in the more efficient suppression of shedding and an earlier reduction in transmission. Serology revealed low-level exposure that was not detectable by RT-qPCR without a more regular sampling schedule, especially after challenge at a younger age.

## 4. Discussion

The present study evaluated the efficacy of a recombinant herpesvirus of turkey–vectored Newcastle disease vaccine (rHVT-ND vaccine), applied alone or in combination with live ND vaccines to commercial layer chickens carrying MDA to NDV, with particular emphasis on early immunity, virus shedding, and transmission dynamics under controlled experimental conditions. The results demonstrated that although all vaccination strategies provided a high level of clinical protection, challenge infection at an early age (3 weeks) still resulted in 5–15% mortality with shedding and transmission to contact birds no matter what vaccination regime was used. However, important differences were observed in the kinetics of immunity, levels of challenge virus shedding, and control of challenge virus transmission, particularly in the first 3–4 weeks of life.

A key finding of this study is that clinical protection alone is not sufficient to characterize vaccine performance, as substantial differences were observed in challenge virus shedding and transmission despite similarly high clinical protection (i.e., high survival rate without ND-related clinical signs). This is consistent with previous studies that have demonstrated that rHVT-ND vaccines can effectively protect against disease but may allow some replication of the challenge virus mainly in the upper respiratory mucosa and at a lower degree in the gut, especially during the early post-vaccination period [15,26,27]. In the present study, this was reflected in the higher frequency of detectable NDV RNA both in the swab samples and by the triggering of the booster effect on the antibody titer against NDV following challenge at a young age (3 weeks of age), especially in birds receiving only the rHVT-ND vaccine.

Completion of the rHVT-ND-based vaccination regimen with the addition of live ND vaccines resulted in a markedly improved early immune response, as evidenced by earlier seroconversion, and significantly enhanced suppression of challenge virus shedding after infection at 3 and 4 weeks of age. This effect can be attributed to the induction of local mucosal immunity by live vaccines, which complements the systemic immunity elicited by the vector vaccine [20]. The importance of mucosal immunity in controlling NDV replication at the primary sites of infection has been well documented [28,29,30]. The early development of mucosal immunity and suppression of virus shedding are particularly relevant under conditions of high infection pressure, where field challenge with the velogenic NDV frequently occurs as early as 2–3 weeks of age.

The booster vaccination applied at 12 days of age further enhanced the development of early immunity to ND, resulting in the most pronounced suppression of virus shedding and reduced detection of challenge virus transmission to contact birds already at 3 weeks of age. This finding highlights the importance of a combined vaccination strategy and supports the concept that supplementing rHVT-ND vaccination with the administration of a live ND vaccine at day old, preferably in the hatchery at hatch, can enhance early protection and improve overall immunity against Newcastle disease during the critical early life period. The prime-boost vaccination regimen compensated for the lag phase associated with vector vaccine-induced immunity.

Another significant contribution of this study is the integrated evaluation of transmission, based on combined analyses of challenge virus shedding and serological results. While RT-qPCR analysis of swab samples at 5 and 14 dpch indicated minimal or absent virus shedding by vaccinated contact birds, serological data revealed that low-level exposure still occurred, especially following an early challenge date (3 weeks of age). This demonstrated that reliance on RT-qPCR detection alone may underestimate transmission events in cases where swab samples are collected a few dates only, or under field conditions when the date of exposure is not known.

The observed discrepancy between the RT-qPCR and serological findings in contact birds, particularly at an early age, is also in line with previous observations indicating that low-level exposure may not always be detectable by RT-qPCR alone. When vaccinated birds are newly exposed to an NDV, viral loads may be too low to result in a positive RT-qPCR result, even though the host’s immune system has already begun producing antibodies [31]. This highlights the importance of using multiple evaluation approaches when assessing vaccine efficacy against transmission.

It should, however, be noted that continuous monitoring of infection status was not performed; therefore, infection dynamics could not be directly assessed. Instead, the infection status of contact birds could only be inferred from their serological responses. Seroconversion in contact birds provided a surrogate estimate of protection against infection and transmission; this should, however, not be interpreted as direct evidence that infection was completely prevented in all protected birds, and the possibility of undetected transient infections cannot be fully excluded.

The transmission model used in this study, involving co-mingling of directly challenged and contact vaccinated birds at a 2:1 ratio, represents a stringent experimental structure with high infection pressure. Under these conditions, all non-vaccinated contact birds became infected and succumbed to ND, confirming the efficient transmission of the challenge virus. In contrast, all vaccination strategies tested were able to prevent the transmission of the challenge virus in an amount that could have led to clinical disease in the vaccinated contact birds. Importantly, combined vaccination programs resulted in earlier and higher reductions in horizontal NDV transmission, as reflected by the absence of both NDV RNA detection and significant antibody responses in the contact birds. Nevertheless, the frequency of post-challenge samplings did not allow us to perform R0 calculation.

The temporal dynamics of shedding and transmission observed in this study are particularly relevant for field conditions. At 3 weeks of age, during the early phase of immunity when birds were developing active immunity in the presence of maternally derived antibodies, the differences between vaccination strategies were most pronounced. Combined vaccination programs significantly reduced both challenge virus shedding and transmission potential compared to rHVT-ND alone. These differences, however, were reduced over time, and by 6 weeks of age, rHVT-ND alone was sufficient to confer strong protection and interrupt virus transmission. This suggests that the primary advantage of combined vaccination lies in bridging the early immunity gap.

From a practical point of view, these findings have important implications for ND control strategies in areas with high infection pressure or early exposure risk. Vaccination programs that include both rHVT-ND and live ND vaccines may provide superior control by reducing virus circulation within the flock and among flocks as well. Using this vaccination strategy, individual birds are protected and even population-level immunity is achieved that would limit challenge virus transmission.

The findings of the present study are consistent with previous reports demonstrating that recombinant HVT-vectored ND vaccines provide strong clinical protection, but may not completely prevent virus shedding, particularly during the early post-vaccination period [8,19,26,27,29]. Several studies have shown that rHVT-ND vaccination significantly reduces virus shedding following challenge; however, some shedding, especially via the oro-nasal route, can still occur [15,27,32]. In agreement with these observations, the present study also demonstrated detectable virus shedding in the rHVT-ND-only group throughout the tested ages in a downward trend, despite high levels of clinical protection. This supports the concept that systemic immunity alone is insufficient to prevent infection and fully control viral replication at mucosal entry sites. It should, however, be highlighted that our experimental design employed a relatively high challenge dose and a high ratio of infected contact birds, which may not fully reflect typical field conditions.

Previous studies evaluating combined vaccination strategies reported improved early protection and reduced virus shedding when live vaccines were administered alongside with rHVT-ND [13,16,33]. The current results extend these findings by demonstrating that such combined approaches not only reduce shedding but also significantly limit transmission to vaccinated contact birds under high-infection-pressure conditions after the complete development of immunity. Importantly, while earlier studies have primarily focused on virus shedding by directly challenged birds, data on transmission dynamics in and within vaccinated flocks remain limited [34]. The present study demonstrated that combined vaccination strategies can effectively interrupt transmission, as evidenced by the absence of detectable NDV RNA at the tested time points and no or a minimal increase in NDV-specific antibody titers in vaccinated contact birds after co-mingling with vaccinated, challenged birds.

Overall, our results confirm and extend previous findings by demonstrating that the primary advantage of combining rHVT-ND with live ND vaccines in a vaccination regimen lies in their ability to enhance the development of earlier immunity, improve the reduction in challenge virus shedding, and limit transmission into and within flocks.

## 5. Conclusions

In conclusion, both rHVT-ND vaccination alone and combined vaccination strategies with live ND vaccines provided high levels of clinical protection against challenge with a velogenic genotype VII.2 NDV strain in commercial layer chickens. Combined vaccination programs, however, offered significant advantages: (i) triggering an earlier onset of protective immunity; (ii) suppressing the velogenic ND challenge virus shedding more effectively; and (iii) improving the control of challenge virus transmission.

These benefits were most evident at 3 and 4 weeks of age (early phase in the development of active immunity), when birds are more endangered if a field virus is introduced to the flock. By 6 weeks of age, differences between vaccination strategies were less apparent, with all vaccinated groups demonstrating complete protection against clinical disease; however, based on the shedding and serology results, combined vaccination regimes showed better results in preventing estimated transmission (80–90%) compared to the rHVT-ND-only program (60%).

The integrated analysis of challenge virus shedding and serological results revealed that even in the absence of detectable virus shedding by RT-qPCR (at two sampling dates), low-level transmission and exposure of contact birds may occur, emphasizing the importance of the use of comprehensive evaluation approaches when assessing virus transmission.

Overall, the results support the use of combined vaccination strategies to enhance the development of early immunity and reduce the spread of NDV in a flock or within flocks, thereby contributing to improved control of Newcastle disease in commercial poultry production, especially in Newcastle disease-endemic geographic areas. Nevertheless, the successful translation of these experimental results into field performance depends on proper vaccine application both at the hatchery and farm levels. Although the present study demonstrated significant reduction in NDV transmission under controlled experimental conditions, further investigations are needed to confirm these results under field conditions and to assess their generalizability to other chicken breeds, production systems, and NDV genotypes.

## Figures and Tables

**Table 1:** Trial design.

Bird Type		Vaccination(s)			Sampling for Vaccine-Take	Serological Monitoring		Velogenic NDV (GVII.2) Challenge			Post-Challenge Samplings
	Group	rHVT-ND (at D0)	Live ND (at D0)	Live ND (at D12)	Spleen and Feather Pulp (w3)	Blood Samplings (w2, w3, w4, w5, w6, w8)	Sub- Group	w3	w4	w6	After All the Challenge Dates; All Groups
Commercial layer (MDA pos)	G1	no	No	no	*n* = 10	*n* = 20	Seeder	*n* = 20	*n* = 20	*n* = 20	5 dpch and 14 dpch: swab samplings 14 dpch blood sampling 1–14 dpch: clinical observations (*n* = all seeder and contact chickens)
							Contact	*n* = 10	*n* = 10	*n* = 10	
	G2	yes	No	no	*n* = 10	*n* = 20	Seeder	*n* = 20	*n* = 20	*n* = 20	
							Contact	*n* = 10	*n* = 10	*n* = 10	
	G3	yes	Enterotropic strain	no	*n* = 10	*n* = 20	Seeder	*n* = 20	*n* = 20	*n* = 20	
							Contact	*n* = 10	*n* = 10	*n* = 10	
	G4	yes	HB 1 strain	no	*n* = 10	*n* = 20	Seeder	*n* = 20	*n* = 20	*n* = 20	
							Contact	*n* = 10	*n* = 10	*n* = 10	
	G5	yes	Enterotropic strain	LaSota strain	*n* = 5	*n* = 20	Seeder	*n* = 20	*n* = 20	*n* = 20	
							Contact	*n* = 10	*n* = 10	*n* = 10	
SPF layer (MDA neg)	G6	no	No	no	no	no	Seeder	*n* = 10	n.a.	n.a.	

Abbreviations: G = group, D = days of age, w = weeks of age; n.a. = not applicable.

**Table 2:** Detection of rHVT-ND vaccine virus at 21 days of age in feather pulp and spleen samples.

Group	Vaccination			rHVT-ND Detection (Positives/Tested)		
	D0	D0	D12	D21		
	rHVT-ND	Live ND	Live ND	Feather Tip	Spleen	Ratio of Positive Birds
G1	no	no	no	0/5	0/5	0%
G2	yes	no	no	10/10	10/10	100%
G3	yes	Enterotropic ND vaccine	no	9/10	10/10	100%
G4	yes	HB 1 vaccine	no	9/10	9/10	100%
G5	yes	Enterotropic ND vaccine	LaSota vaccine	9/10	9/10	100%

**Table 3:** Clinical protection rates after vNDV challenges at different ages. The corresponding 95% confidence intervals are shown in brackets.

Seeder (Direct Challenged) Subgroups	Challenge 1 (w3)	Challenge 2 (w4)	Challenge 3 (w6)
Unvaccinated control	0% (0–16.1%)	0% (0–16.1%)	0% (0–16.8%)
rHVT-ND vaccine	85% (64–94.8%)	85% (64–94.8%)	100% (83.9–100%)
rHVT-ND and Enterotropic ND vaccine	90% (69.9–98.2%)	100% (83.9–100%)	100% (83.2–100%)
rHVT-ND and HB1 vaccine	95% (76.4–99.7%)	100% (83.9–100%)	100% (83.9–100%)
rHVT-ND and Enterotropic ND and LaSota vaccine	95% (76.4–99.7%)	100% (83.2–100%)	100% (83.9–100%)
**Contact subgroups**			
Unvaccinated control	0% (0–27.8%)	0% (0–27.8%)	0% (0–27.8%)
rHVT-ND vaccine	100% (72.2–100%)	100% (72.2–100%)	100% (72.2–100%)
rHVT-ND and Enterotropic ND vaccine	100% (72.2–100%)	100% (72.2–100%)	100% (72.2–100%)
rHVT-ND and HB1 vaccine	100% (72.2–100%)	100% (72.2–100%)	100% (72.2–100%)
rHVT-ND and Enterotropic ND and LaSota vaccine	100% (72.2–100%)	100% (72.2–100%)	100% (72.2–100%)

**Table 4:** Proportion of samples with detectable NDV RNA in the oronasal and cloacal swabs in seeder birds at 5 dpch. The corresponding 95% confidence intervals are shown in brackets.

Seeder (Direct Challenged) Chickens	Challenge 1 (w3)		Challenge 2 (w4)		Challenge 3 (w6)	
	ON swab	CLO swab	ON swab	CLO swab	ON swab	CLO swab
Unvaccinated control	100% (83.9–100%)	100% (83.9–100%)	100% (83.9–100%)	100% (83.9–100%)	100% (83.2–100%)	100% (83.2–100%)
rHVT-ND vaccine	95% (76.4–99.7%)	80% (58.4–91.9%)	85% (64–94.8%)	45% (25.8–65.8%)	25% (11.2–46.9%)	0% (0.0–16.1%)
rHVT-ND & Enterotropic ND vaccine	55% (34.2–74.2%)	30% (14.5–51.9%)	0% (0–16.1%)	0% (0–16.1%)	42% (23.1–63.7%)	0% (0.0–16.8%)
rHVT-ND & HB1 vaccine	50% (29.9–70.1%)	35% (18.1–56.7%)	0% (0–16.1%)	0% (0–16.1%)	25% (11.2–46.9%)	5% (0.3–23.6%)
rHVT-ND & Enterotropic ND and LaSota vaccine	80% (58.4–91.9%)	30% (14.5–51.9%)	15% (5.5–37.6%)	5% (0.3–23.6%)	5% (0.3–24.6%)	0% (0.0–16.8%)

Abbreviations: ON swab = oro-nasal (choanal slit) swab, CLO swab = cloacal swab.

**Table 5:** Mean HI antibody titres (±SD) in the different vaccination groups before and after challenge at 3, 4, and 6 weeks of age in seeder birds, contact birds, and age-matched non-challenged groupmates.

Vaccination	rHVT-ND Vaccine		rHVT-ND and Enterotropic ND Vaccine		rHVT-ND and HB1 Vaccine		rHVT-ND and Enterotropic and LaSota Vaccine	
	Challenge 1 (w3)							
Subgroup	pre-ch.	post-ch.	pre-ch.	post-ch.	pre-ch.	post-ch.	pre-ch.	post-ch.
Non-challenged	1.9	3.3	3.1	4.9	3.0	3.8	3.5	5.7
	±1.0	±1.0	±1.8	±1.7	±1.0	±1.2	±1.3	±1.3
Seeder	1.9	11.0	3.1	11.0	3.0	11.0	3.5	9.8
	±1.0	±0.9	±1.8	±1.4	±1.0	±1.5	±1.3	±2.7
Contact	2.0	11.7	2.6	9.7	3.2	11.9	3.4	7.7
	±0.8	±0.5	±0.9	±2.5	±1.0	±0.3	±0.9	±2.8
	Challenge 2 (w4)							
Subgroup	pre-ch.	post-ch.	pre-ch.	post-ch.	pre-ch.	post-ch.	pre-ch.	post-ch.
Non-challenged	2.3	3.9	4.8	5.1	4.2	4.1	4.8	5.3
	±0.8	±1.1	±1.6	±0.9	±1.4	±0.9	±1.5	±0.9
Seeder	2.3	9.3	4.8	8.3	4.2	6.0	4.8	6.4
	±0.8	±1.0	±1.6	±1.6	±1.4	±1.4	±1.5	±1.8
Contact	2.4	9.4	5.3	6.8	4.2	4.4	4.7	4.8
	±0.5	±1.0	±1.8	±2.6	±0.7	±0.8	±1.0	±0.9
	Challenge 3 (w6)							
Subgroup	pre-ch.	post-ch.	pre-ch.	post-ch.	pre-ch.	post-ch.	pre-ch.	post-ch.
Non- challenged	3.9	5.0	5.1	5.6	4.1	5.2	5.3	5.3
	±1.1	±1.2	±0.9	±1.0	±0.9	±0.8	±0.9	±0.6
Seeder	3.9	6.1	5.5	6.8	4.3	6.8	5.2	5.3
	±1.1	±1.1	±0.9	±1.1	±1.0	±1.1	±1.2	±1.2
Contact	3.8	5.3	4.3	4.5	3.8	4.5	5.5	4.4
	±0.8	±1.2	±1.5	±1.1	±0.7	±0.6	±0.9	±0.4

Abbreviations: SD = standard deviation, pre-ch. = pre-challenge, post-ch. = post-challenge.

**Table 6:** Evaluation of infection status in contact subgroups after challenges at 3, 4 and 6 weeks of age. The corresponding 95% confidence intervals are shown in brackets.

	Ratio of Negative Chickens Based on the RT-qPCR *				No/Low Anti-NDV HI Antibody Response **	Ratio of Birds Protected Against NDV Infection
	5 dpch		14 dpch			
	ON swab	CLO swab	ON swab	CLO swab		
**Ch1 (w3)**						
Unvaccinated control	30% (10.8–60.3%)	10% (0.5–40.4%)	n.a.	n.a.	n.a.	0% (0–27.8%)
rHVT-ND vaccine	30% (10.8–60.3%)	70% (39.7–89.2%)	40% (16.8–68.7%)	60% (31.3–83.2%)	0% (0–27.8%)	0% (0–27.8%)
rHVT-ND & Enterotropic ND vaccine	70% (39.7–89.2%)	100% (72.2–100%)	80% (49–96.4%)	100% (72.2–100%)	0% (0–27.8%)	0% (0–27.8%)
rHVT-ND & HB1 vaccine	70% (39.7–89.2%)	90% (59.6–99.5%)	70% (39.7–89.2%)	80% (49–96.4%)	0% (0–27.8%)	0% (0–27.8%)
rHVT-ND & Enterotropic ND & LaSota vaccine	60% (31.3–83.2%)	90% (59.6–99.5%)	100% (72.2–100%)	100% (72.2–100%)	40% (16.8–68.7%)	20% (3.6–51%)
**Ch2 (w4)**						
Unvaccinated control	0% (0–27.8%)	30% (10.8–60.3%)	n.a.	n.a.	n.a.	0% (0–27.8%)
rHVT-ND vaccine	90% (59.6–99.5%)	100% (72.2–100%)	90% (59.6–99.5%)	100% (72.2–100%)	0% (0–27.8%)	0% (0–27.8%)
rHVT-ND & Enterotropic ND vaccine	90% (59.6–99.5%)	100% (72.2–100%)	90% (59.6–99.5%)	100% (72.2–100%)	60% (31.3–83.2%)	50% (23.7–76.3%)
rHVT-ND & HB1 vaccine	100% (72.2–100%)	100% (72.2–100%)	78% (45.3–96.1%)	100% (71.1–100%)	100% (71.1–100%)	78% (45.3–96.1%)
rHVT-ND & Enterotropic ND & LaSota vaccine	90% (59.6–99.5%)	90% (59.6–99.5%)	90% (59.6–99.5%)	100% (72.2–100%)	90% (59.6–99.5%)	60% (31,3–83,2%)
**Ch3 (w6)**						
Unvaccinated control	0% (0–27.8%)	10% (0.5–40.4%)	n.a.	n.a.	n.a.	0% (0–27.8%)
rHVT-ND vaccine	100% (72.2–100%)	90% (59.6–99.5%)	80% (49–96.4%)	70% (39.7–89.2%)	90% (59.6–99.5%)	60% (31.3–83.2%)
rHVT-ND & Enterotropic ND vaccine	80% (49–96.4%)	100% (72.2–100%)	100% (72.2–100%)	100% (72.2–100%)	100% (72.2–100%)	80% (49–96.4%)
rHVT-ND & HB1 vaccine	100% (72.2–100%)	90% (59.6–99.5%)	90% (59.6–99.5%)	100% (72.2–100%)	90% (59.6–99.5%)	70% (39.7–89.2%)
rHVT-ND & Enterotropic ND & LaSota vaccine	90% (59.6–99.5%)	100% (72.2–100%)	100% (72.2–100%)	100% (72.2–100%)	100% (72.2–100%)	90% (59.6–99.5%)

Note: * = Protected if NDV was not detectable (the allocated Ct value is 40; ** = if the increase in the log_2_ HI titer during the 14-day post-challenge period is less than or equal to 2. Abbreviation: n.a. = not applicable.

## Data Availability

The individual raw data are provided in the supplementary tables (Tables S1–S4).

## Supplementary Materials

The following supporting information can be downloaded at https://www.mdpi.com/article/10.3390/vaccines14100859/s1: Table S1: Individual data of the challenge 1 at 3 weeks of age; Table S2: Individual data of the challenge 2 at 4 weeks of age; Table S3: Individual data of the challenge 3 at 6 weeks of age; Table S4: Individual data ofserological monitoring of non-challenged animals.

## Abbreviations

The following abbreviations are used in this manuscript:

vNDV	velogenic Newcastle Disease Virus
ON	Oronasal
CLO	Cloacal
SPF	specified pathogen-free
Ch	Challenge
PFU	plaque forming unit
rHVT-ND	recombinant herpesvirus of turkeys-vectored Newcastle disease vaccine
PBS	phosphate-buffered saline
MDA	maternally derived antibodies
EID_50_	50% Embryo Infective Dose
n.a.	not applicable
D	day of age
w	week of age
SD	standard deviation
